# Diagnostic Accuracy of Intraoperative Neurophysiologic Monitoring in Spinal Deformity Surgery: Systematic Review and Meta-Analysis

**DOI:** 10.1177/21925682261492292

**Published:** 2026-09-28

**Authors:** Brendan T. O’Reilly, Alexandra M. Dunker, Drue M. Domagala, Allie C. Reynolds, Catalina Garcia, Sohan Arakal, Giselle K. Henry, David Travis Johnston, Rajiv S. Dharnipragada, Stavros Matsoukas, Theodore Gallas, Noah C. Fulcomer, Shyon K. Ahmadi, Alexander Lee, Eric O. Klineberg

**Affiliations:** 1 Department of Orthopedic Surgery, McGovern Medical School, 12340 The University of Texas Health Science Center at Houston (UTHealth), Houston, TX, USA; 2 3341 University of Arkansas, Fayetteville, AR, USA; 3 3990 Rice University, Houston, TX, USA; 4 Department of Neurosurgery, McGovern Medical School, The University of Texas Health Science Center at Houston (UTHealth), Houston, TX, USA

**Keywords:** intraoperative neuromonitoring, intraoperative neurophysiologic monitoring, IONM, spinal deformity surgery, scoliosis, kyphosis, motor evoked potentials, somatosensory evoked potentials, MEP, SSEP, diagnostic accuracy, sensitivity, specificity, meta-analysis, systematic review

## Abstract

**Study Design:**

Systematic review and diagnostic accuracy meta-analysis.

**Objective:**

To evaluate the diagnostic accuracy of intraoperative neurophysiologic monitoring (IONM) for detecting postoperative neurologic deficits in spinal deformity surgery and assess performance across monitoring modalities.

**Methods:**

A systematic review of PubMed, Embase, and Web of Science (2000-2026) was performed in accordance with PRISMA guidelines. Studies reporting intraoperative neuromonitoring and postoperative neurologic outcomes were included. Diagnostic accuracy metrics were calculated from contingency tables and pooled using random-effects models. Subgroup analyses were performed by monitoring modality.

**Results:**

A total of 43 studies were included in the systematic review. Of these, 38 had constructable 2x2 tables and contributed to the specificity and false-positive rate analyses, while 36 contributed to the sensitivity, false-negative rate, and diagnostic-odds ratio models. Pooled sensitivity was 90.1% (95% CI, 83.8-94.2%), and pooled specificity was 95.4% (95% CI, 93.0-97.0%). The diagnostic odds ratio was 206.8 (95% CI, 92.8-460.9). Heterogeneity was observed (I^2^ = 56.7% for sensitivity and 95.3% for specificity). Sensitivity and specificity remained broadly comparable across conventional monitoring modalities, and between-modality differences were not statistically significant.

**Conclusions:**

IONM demonstrates high specificity and strong sensitivity for detecting neurologic injury in spinal deformity surgery. Performance was similar across conventional modalities, although sensitivity and false-negative rates varied across studies. These findings support the continued use of IONM as an intraoperative safety tool while highlighting the need for standardized reporting and improved interpretation of monitoring signals.

## Introduction

Neurologic injury remains one of the most impactful complications of spinal deformity surgery, with risks occurring during deformity correction maneuvers, prolonged operative times, instrumentation placement, and manipulation of the neural elements including spinal cord and nerve roots. Although modern surgical techniques and perioperative care have improved safety, postoperative neurologic deficits, including spinal cord injury, myelopathy, focal motor deficits, and radiculopathy, continue to occur and may result in significant functional morbidity.^1-3^ As a result, intraoperative neurophysiologic monitoring (IONM) has become widely adopted as an adjunct intended to improve neurologic safety through continuous real-time assessment of neural pathway integrity during surgery.

IONM encompasses several modalities targeting different neuroanatomical pathways, most commonly somatosensory evoked potentials (SSEPs), transcranial motor evoked potentials (MEPs), and electromyography (EMG). SSEPs primarily evaluate dorsal column sensory pathways, whereas MEPs assess corticospinal tract function and provide dynamic feedback regarding motor pathway integrity. EMG monitoring may detect nerve root irritation or pedicle screw malposition. Multimodal monitoring strategies combining these techniques have increasingly been favored because they enhance sensitivity to both ascending and descending pathway compromise.^4,5^ Clinical studies and practical reviews have suggested that multimodal IONM provides improved diagnostic coverage compared with single-modality approaches, particularly during complex, cord-level deformity correction.^6,7^

Despite widespread utilization, the true diagnostic accuracy of IONM remains controversial. Systematic reviews have demonstrated variability in reported sensitivity and specificity depending on monitoring modality, alert thresholds, surgical population, and outcome definitions.^8,9^ Some studies report high sensitivity and negative predictive value, supporting the role of IONM as an early warning system capable of preventing irreversible neurologic injury through intraoperative intervention.^10^ However, others highlight challenges including false-positive alerts, anesthetic-related signal changes, and inconsistent correlation between signal loss and clinically meaningful deficits.^11,12^ This variability reflects heterogeneity in monitoring protocols, differences in surgeon response algorithms, and inconsistent definitions of neurologic outcomes across the literature.

A critical limitation of prior research is the frequent aggregation of neurologic complications into a single outcome category, despite important differences between spinal cord–level injury and root-level or lower motor neuron (LMN) deficits. Cord-level injuries typically manifest as diffuse or bilateral motor and sensory deficits, or changes in ASIA grade, reflecting compromise of long-tract pathways. In contrast, root-level deficits such as postoperative C5 palsy, new radiculopathy, or focal myotomal weakness may arise from traction, foraminal narrowing, positioning, or direct nerve root irritation rather than spinal cord ischemia or injury. Because different IONM modalities preferentially assess specific neural structures (e.g., MEPs for corticospinal pathways and EMG for root irritation), their predictive performance may differ depending on the type of neurologic injury. Slow traction injuries may not trigger EMG activity or produce substantial changes in MEP or SSEP signals. Unfortunately, few studies explicitly stratify diagnostic accuracy based on cord versus root-level outcomes, limiting clinical interpretation of IONM alerts.^13,14^

Additionally, many available studies are limited by small sample sizes, single-center design, or retrospective methodology, which complicates interpretation of rare events such as permanent neurologic injury. Differences in patient populations, deformity severity, surgical technique, and monitoring thresholds further contribute to heterogeneity. Emerging work exploring risk prediction models and deformity characteristics associated with IONM signal changes underscores the complexity of interpreting monitoring data and highlights the need for pooled analyses to generate more precise estimates of diagnostic performance.^15^ Diagnostic accuracy meta-analysis is particularly suited to this context because it enables aggregation of sensitivity, specificity, and diagnostic odds ratios across heterogeneous datasets while accounting for between-study variability.

Moreover, the expanding use of IONM across spine surgery has raised questions regarding its optimal indications, cost-effectiveness, and role in improving patient outcomes. While some studies support routine use in high-risk deformity procedures,^16,17^ others suggest that predictive performance may vary depending on monitoring modality or neurologic endpoint, particularly for root-level injuries where signal changes may be less reliable predictors of postoperative deficit. Clarifying these relationships is critical for refining intraoperative decision-making, improving monitoring algorithms, and guiding future guideline development (Table 1).

**Table 1.** Characteristics of Included Studies

First Author (Year)	Sample Size (n)	Monitoring Modality	Study Design	Outcome Assessed
Pelosi L 2002^18^	97	Multimodal MEP + SSEP +/- EMG	Retrospective	Predictive Value
Noonan KJ 2002^19^	134	Multimodal MEP + SSEP +/- EMG	Retrospective	Sensitivity/Specificity
Langeloo DD 2003^20^	145	MEP only	Retrospective	Sensitivity/Specificity
Leung YL 2005^21^	871	SSEP only	Retrospective	Sensitivity/Specificity
Accadbled F 2006^22^	41	Nonstandard evoked-potential technique	Retrospective	Sensitivity/Specificity
Eggspuehler 2007^23^	217	Multimodal MEP + SSEP +/- EMG	Prospective	Sensitivity/Specificity
Lieberman JA 2008^24^	35	MEP only	Retrospective	Sensitivity/Specificity
Master DL 2008^25^	8	SSEP only	Retrospective	Neurologic Recovery
Hsu B 2008^26^	144	MEP only	Prospective	Sensitivity/Specificity
Quraishi NA 2009^27^	102	Multimodal MEP + SSEP +/- EMG	Retrospective	Sensitivity/Specificity
Kundnani VK 2010^28^	354	Multimodal MEP + SSEP +/- EMG	Prospective	Sensitivity/Specificity
Park P 2011^29^	29	MEP only	Retrospective	Sensitivity/Specificity
Feng B 2012^30^	176	Multimodal MEP + SSEP +/- EMG	Retrospective	Sensitivity/Specificity
Muchow RD 2013^31^	222	Multimodal MEP + SSEP +/- EMG	Retrospective	Sensitivity/Specificity
Thirumala PD 2014^32^	477	SSEP only	Retrospective	Detection Rate
Zhuang Q 2014^33^	1,162	MEP only	Retrospective	Sensitivity/Specificity
Bhagat S 2015^34^	351	Multimodal MEP + SSEP +/- EMG	Retrospective	Other
Wang S 2016^35^	118	MEP only	Retrospective	Sensitivity/Specificity
Chang SH 2016^36^	190	Multimodal MEP + SSEP +/- EMG	Prospective	New Motor Deficit
Gavaret M 2016^37^	78	Nonstandard evoked-potential technique	Retrospective	Sensitivity/Specificity
Neira VM 2016^38^	275	Multimodal MEP + SSEP +/- EMG	Retrospective	New Motor Deficit
Wang S 2017^39^	332	MEP only	Retrospective	New Motor Deficit
Acharya S 2017^40^	61	MEP only	Retrospective	Sensitivity/Specificity
Ushirozako H 2018^13^	295	SSEP only	Retrospective	Detection Rate
Kobayashi K 2018^41^	83	Multimodal MEP + SSEP +/- EMG	Retrospective	Sensitivity/Specificity
Miller SM 2019^42^	125	MEP only	Retrospective	Predictive Value
Ushirozako H 2019^11^	393	MEP only	Retrospective	New Motor Deficit
Lau D 2019^43^	242	MEP only	Retrospective	New Motor Deficit
Lieberman JA 2019^44^	542	MEP only	Retrospective	Sensitivity/Specificity
Tsirikos AI 2020^7^	1,155	Multimodal MEP + SSEP +/- EMG	Retrospective	Detection Rate
Takahashi M 2021^45^	1,934	MEP only	Prospective	Sensitivity/Specificity
Rachmat O 2021^46^	93	MEP only	Retrospective	Sensitivity/Specificity
Huang X 2022^47^	68	Multimodal MEP + SSEP +/- EMG	Retrospective	Sensitivity/Specificity
Qiu 2022^48^	87	Multimodal MEP + SSEP +/- EMG	Retrospective	Sensitivity/Specificity
Kerdoncuff A 2023^49^	190	MEP only	Retrospective	Sensitivity/Specificity
Troyer SC 2023^50^	14	DNEP-focused	Retrospective	Sensitivity/Specificity
Deng Y 2024^51^	180	DNEP-focused	Retrospective	Sensitivity/Specificity
Ali S 2024^52^	170	Multimodal MEP + SSEP +/- EMG	Retrospective	Sensitivity/Specificity
Ma N 2024^53^	399	Multimodal MEP + SSEP +/- EMG	Retrospective	Sensitivity/Specificity
Hardy S 2025^54^	62	Multimodal MEP + SSEP +/- EMG	Retrospective	Sensitivity/Specificity
Chen 2025^55^	103	MEP only	Retrospective	Sensitivity/Specificity
Kamenova 2025^56^	155	Multimodal MEP + SSEP +/- EMG	Retrospective	Sensitivity/Specificity
Chen M 2026^57^	443	Multimodal MEP + SSEP +/- EMG	Retrospective	Neurologic complication incidence

***Note.*** Monitoring modality reflects the primary intraoperative neurophysiologic monitoring approach reported or used for diagnostic data extraction. Multimodal monitoring refers to combined MEP and SSEP monitoring with or without EMG. DNEP-focused and other nonstandard evoked-potential approaches are labeled separately. Outcome assessed reflects the primary diagnostic or clinical endpoint reported in each study and used for data extraction.

**Abbreviations:** IONM, intraoperative neurophysiologic monitoring; MEP, motor evoked potentials; SSEP, somatosensory evoked potentials; DNEP, descending neurogenic evoked potential.

This systematic review and meta-analysis evaluates the diagnostic accuracy of intraoperative neurophysiologic monitoring in spinal deformity surgery using pooled estimates of sensitivity, specificity, and diagnostic odds ratios derived from random-effects modeling. By synthesizing available evidence, this study aims to provide a more comprehensive understanding of how intraoperative monitoring performance translates to postoperative neurologic outcomes in the available published data. Additionally, subgroup analyses by monitoring modality were performed to better characterize variability in diagnostic performance. Through addressing heterogeneity across studies, this analysis seeks to establish a clearer framework for interpreting IONM alerts and optimizing neurologic safety in complex spine surgery.

## Methods

### Study Design and Reporting Standards

This study was conducted in accordance with the Preferred Reporting Items for Systematic Reviews and Meta-Analyses (PRISMA 2020) guidelines. The goal of this meta-analysis was to evaluate the performance of IONM systems during spinal surgery in detecting postoperative neurologic deficits.

A comprehensive literature search was performed to identify relevant studies published between January 1, 2000, and May 1, 2026, using PubMed, Embase and Web of Science. Search terms included combinations of intraoperative monitoring modalities including MEPs, SSEPs, and IONM, as well as spinal deformity conditions such as scoliosis and kyphosis. Diagnostic accuracy terms including sensitivity, specificity, accuracy, and predictive value were also included. The search strategy incorporated both Medical Subject Headings (MeSH) and free-text terms. The full search query is provided in the Supplemental Material.

### Study Selection

Three databases were searched, including PubMed, Embase, and Web of Science. Studies were eligible for inclusion if they (1) evaluated IONM during spinal deformity surgery and (2) reported postoperative neurologic outcomes. Additionally, studies were required to provide sufficient data to either derive diagnostic accuracy metrics or allow for reconstruction of required contingency tables. Studies were excluded if they lacked adequate data for diagnostic contingency table construction, did not report relevant postoperative neurologic outcomes, or did not include spinal deformity surgery. When a study included a mixed surgical population, deformity-specific data were used when available. Case reports, review articles, and non-human studies were also excluded.

### Data Extraction

Data were extracted from each included study using a standardized approach, including author, year of publication, sample size, monitoring modality, anesthesia regimen when reported, alert-threshold criteria, and diagnostic data including true positives, false positives, true negatives, and false negatives. When contingency table data were not reported directly, values were reconstructed from the available study data and checked against the original publication. Diagnostic data were extracted using the unit reported by each study, which was most often the patient but in some studies was the procedure or operation.

### Outcome Measures

The primary outcomes of interest were the sensitivity and specificity of IONM for detecting postoperative neurologic deficits. Secondary outcomes included the diagnostic odds ratio, false-negative and false-positive rates, SROC performance, exploratory assessment of small-study effects, and subgroup differences in diagnostic performance across monitoring modalities.

### Statistical Analysis

A diagnostic test accuracy meta-analysis was performed, with sensitivity and specificity calculated for each study using standard definitions. Pooled estimates were generated using random-effects logit models to account for between-study heterogeneity. A continuity correction of 0.5 was applied to zero cells in otherwise estimable 2 × 2 tables. Studies with no postoperative neurologic deficits, defined as TP + FN = 0, were not assigned an artificial sensitivity or false-negative rate and did not contribute to the sensitivity, false-negative rate, or diagnostic odds ratio analyses. Studies with TN + FP = 0 would similarly have been excluded from the specificity and false-positive rate analyses. Proportions were logit-transformed before pooling and back-transformed for reporting. Prediction intervals were calculated to show the expected range of results in a comparable future study.

Meta-regression was used to determine whether conventional monitoring modality accounted for differences in sensitivity and specificity. False-negative rates were calculated only in studies that reported at least one postoperative neurologic deficit, while false-positive rates were calculated only in studies that included patients without a postoperative neurologic deficit. False-negative and false-positive rates were also compared across conventional monitoring modalities.

Statistical heterogeneity was assessed using the I^2^ statistic, and a summary receiver operating characteristic (SROC) curve was constructed to show overall diagnostic performance. Analyses were performed in R using the metafor package, with restricted maximum likelihood estimation for the random-effects models. An exploratory funnel plot of log diagnostic odds ratios was generated to look for possible small-study effects; no formal test for asymmetry was performed.

Subgroup analyses evaluated differences in diagnostic performance across monitoring modalities, including MEP only, SSEP only, and multimodal MEP + SSEP with or without EMG. Studies that primarily used DNEPs or other less commonly used evoked-potential techniques were included in the overall analysis when a 2 × 2 table could be reconstructed. Because these approaches were not directly comparable with the MEP-only, SSEP-only, and multimodal groups, they were reported separately. No included study used EMG as a standalone index test.

Additional analyses were performed to further characterize variability in diagnostic performance across studies. False-negative and false-positive rates were pooled by monitoring modality using random-effects models. These rates were included only when they could be calculated from the reported data. We used meta-regression to compare the conventional monitoring groups. Study-level sensitivity and specificity were plotted by modality, and the relationship between study size and diagnostic performance was examined graphically. Anesthesia regimens and alert thresholds were summarized descriptively because reporting varied across studies and was closely related to monitoring modality and study design.

### Risk of Bias Assessment

Risk of bias was assessed at the individual study level using the Quality Assessment of Diagnostic Accuracy Studies-2 (QUADAS-2) tool. Four domains were evaluated: patient selection, index test (IONM), reference standard (postoperative neurologic outcomes), and flow and timing. Each domain was rated as low, unclear, or high risk based on predefined criteria adapted for IONM studies. Patient selection evaluated the presence of consecutive or representative surgical populations and was considered low risk when the study did so. The index test domain assessed the clarity and consistency of IONM alert definitions and thresholds. The reference standard evaluated the definition and timing of postoperative neurologic assessment. Flow and timing assessed completeness of outcome reporting and consistency of follow-up.

Given variability in reporting and the retrospective design of most included studies, a conservative approach was applied, assigning unclear risk when methodological detail was insufficient. Risk of bias assessments informed interpretation of results but were not used as exclusion criteria.

## Result*s*

### Study Selection

The database search identified 473 records across PubMed, Embase, and Web of Science. After 174 duplicates were removed, 299 records underwent title and abstract screening. Of these, 131 were excluded, leaving 168 reports for full-text review. A total of 125 reports were excluded after full-text review. The remaining 43 studies were included in the systematic review; 38 had constructable contingency tables and were included in the pooled diagnostic accuracy analysis, while five were retained for qualitative synthesis only. The study selection process is illustrated in the PRISMA flow diagram (Figure 1). Of the included studies, anesthesia maintenance could be classified as total intravenous anesthesia (TIVA) in 24 of 43 studies (55.8%), inhalation anesthesia in 2 (4.7%), and mixed or transitioned regimens in 9 (20.9%), with insufficient reporting in 8 studies (18.6%). Of the 38 studies with constructable tables, the corresponding counts were 21, 2, 8, and 7, respectively. Alert threshold reporting was variable where complete signal loss was reported in 22 studies, amplitude-reduction criteria in 40, latency-change criteria in 27, and 1 study had unclear or insufficiently reported threshold criteria.

**Figure 1.**
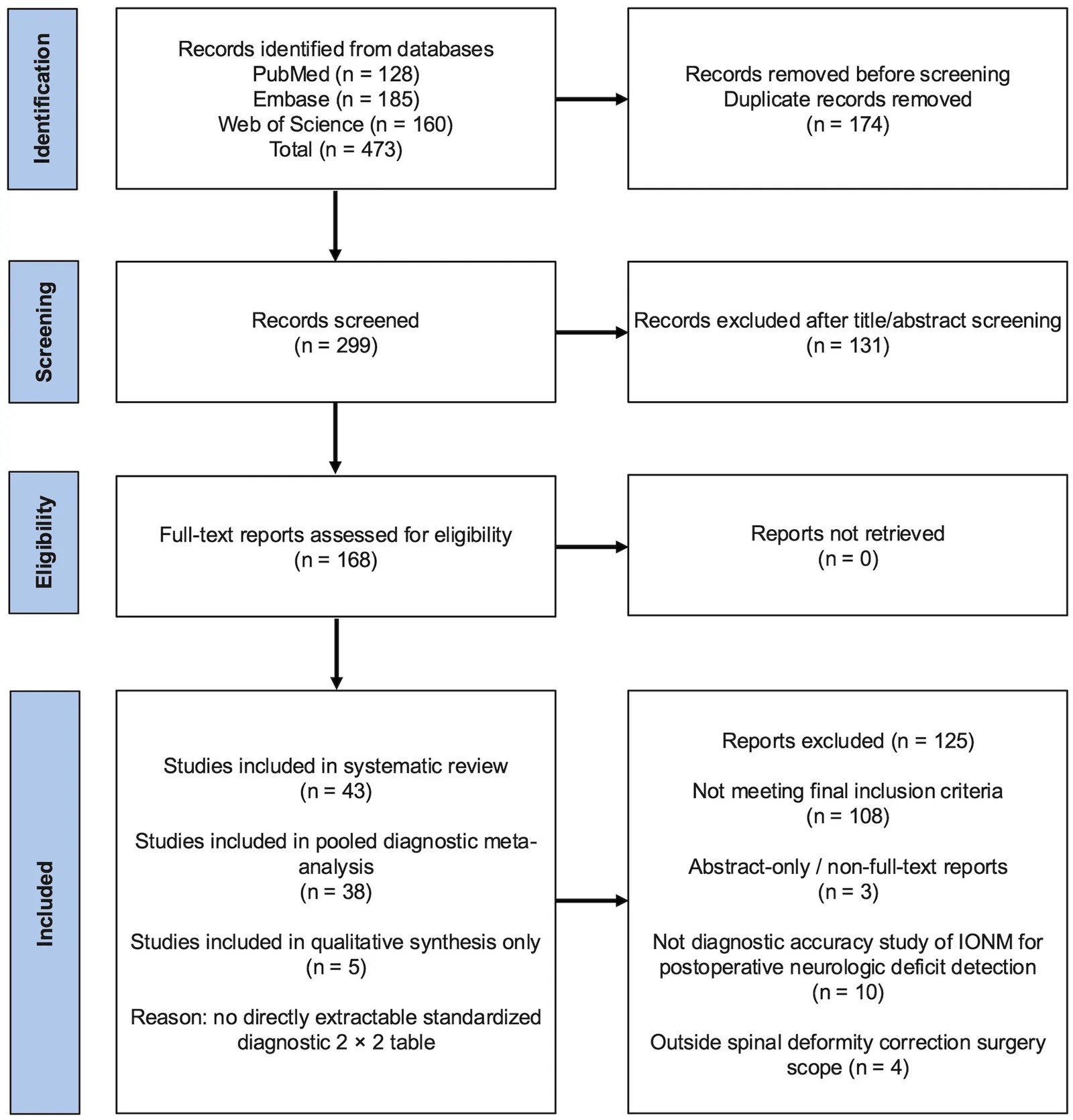
PRISMA 2020 flow diagram of study identification, screening, eligibility assessment, and inclusion. Of 473 records identified, 299 were screened after duplicate removal and 168 full-text reports were assessed. Final inclusion comprised 43 studies in the systematic review, including 38 studies in the pooled diagnostic accuracy meta-analysis and 5 studies retained for qualitative synthesis only

### Risk of Bias Assessment

Risk-of-bias assessment across all 43 included studies showed an overall trend toward low or unclear risk of bias across most QUADAS-2 domains (Figures 2 and 3). When domain-level methodological detail was insufficient or unavailable, studies were conservatively displayed as unclear risk rather than excluded. The main reason for patient selection being rated as unclear risk was limited reporting of consecutive or representative enrollment. The index test domain demonstrated a low risk of bias, since most studies clearly defined intraoperative neuromonitoring modalities and alert criteria, although variability was noted in thresholds. The reference standard domain was frequently rated as unclear risk of bias because of heterogeneity in the definition and timing of post-operative neurological assessment. Inconsistent reporting of the follow-up duration as well as deficits in complete outcome data meant that flow and timing were also given unclear risk ratings.

**Figure 2.**
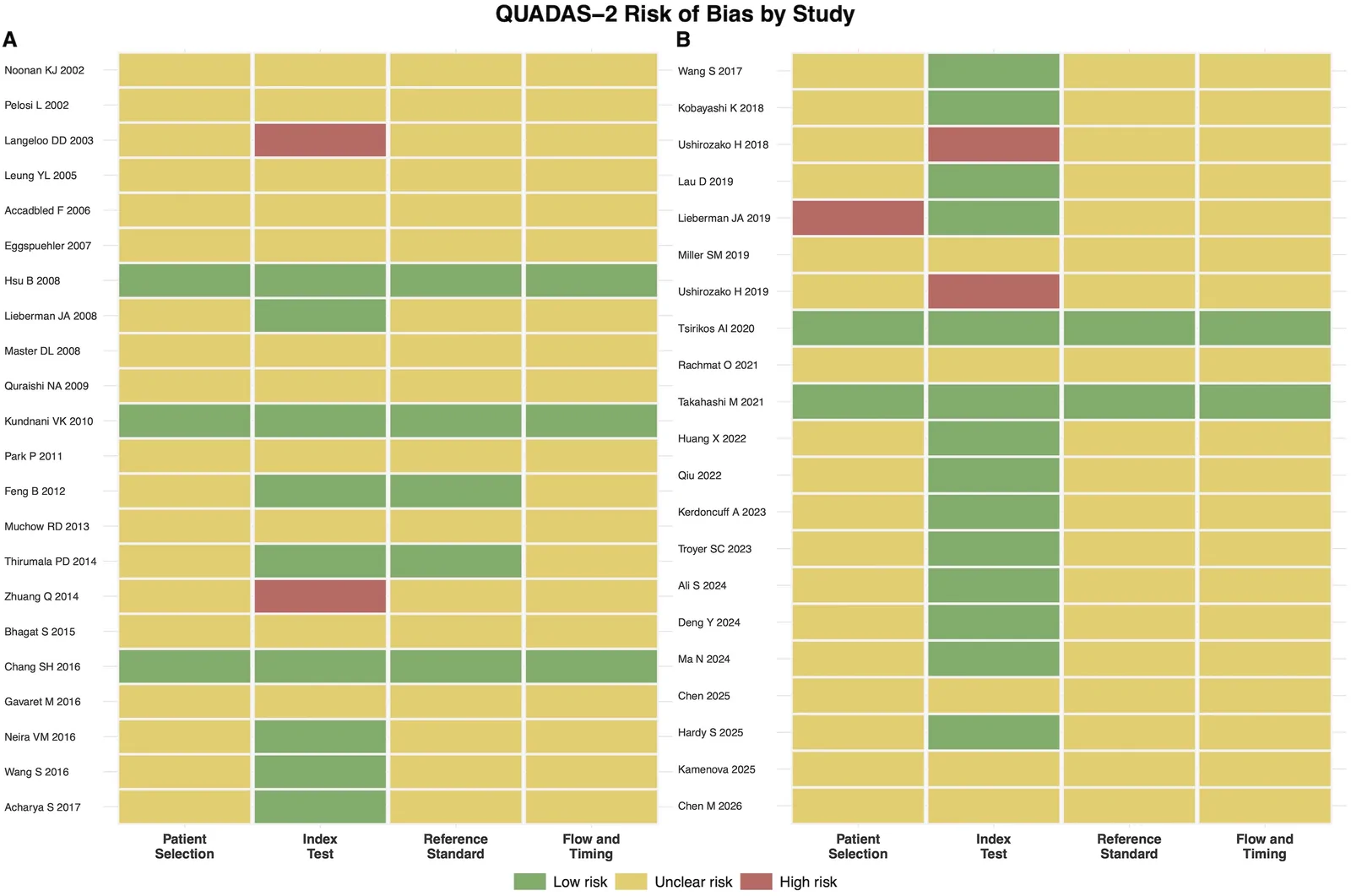
QUADAS-2 risk of bias assessment at the study level across all 43 studies included in the systematic review and qualitative synthesis. (A) First 22 included studies. (B) Remaining 21 included studies. Domains without sufficient source information to permit judgment were coded as unclear risk of bias

**Figure 3.**
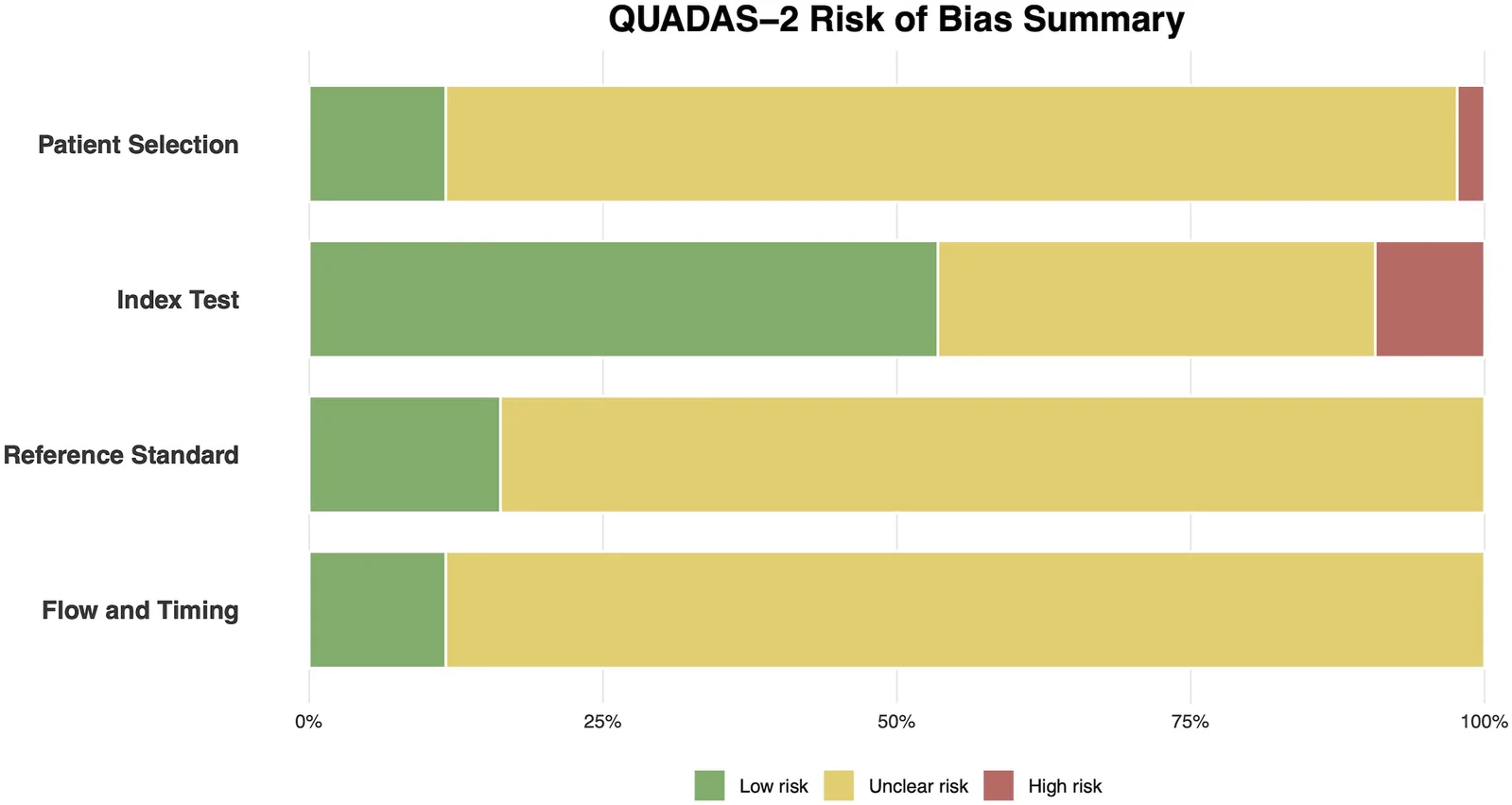
Summary of QUADAS-2 risk of bias across all 43 studies included in the systematic review and qualitative synthesis. Bars represent the proportion of studies rated as low, unclear, or high risk of bias for each domain, including patient selection, index test, reference standard, and flow and timing

Additionally, a smaller proportion of studies had a high risk of bias. The main contributing factors to this were incomplete outcome reporting, non-standardized definitions of neurological deficits, or an unclear inclusion criterion of all monitored patients in the final analyses. Overall, these findings reflect the retrospective review bias as well as variability in reporting practices in the literature. Since there is a predominant rating of low or unclear bias, pooled estimates were interpreted in the context of moderate methodological heterogeneity. The findings suggest that while acceptable methodological quality existed, variability was still a contributing factor to the heterogeneity that was observed.

### Diagnostic Accuracy

Across the included studies, IONM had a pooled sensitivity of 90.1% (95% confidence interval [CI], 83.8-94.2%) and a pooled specificity of 95.4% (95% CI, 93.0-97.0%) for detecting postoperative neurologic deficits. Prediction intervals were wider for both sensitivity (43.4%-99.1%) and specificity (62.1%-99.6%), reflecting substantial variation in performance across study settings. The pooled false-negative rate was 9.9% (95% CI, 5.8-16.2%), while the pooled false-positive rate was 4.6% (95% CI, 3.0-7.0%). Prediction intervals were large for both false-negative rates (0.9%-56.6%), and false-positive rates (0.4%-37.9%) indicating variability between included studies. However, the pooled diagnostic odds ratio was 206.8 (95% CI, 92.8-460.9) further supporting the overall diagnostic performance of IONM.

There was considerable variation across the included studies, with an I^2^ of 56.7% for sensitivity and 95.3% for specificity (Table 2). Sensitivity varied across individual studies (Figure 4), while specificity remained high across most studies (Figure 5). When false-negative and false-positive rates were compared by monitoring modality (Figure 6), the greatest variation was seen in false-negative rates. Meta-regression did not show significant differences between conventional monitoring modalities for sensitivity (QM = 0.206, p = 0.902), specificity (QM = 0.984, p = 0.612), false-negative rate (p = 0.902) or false-positive rate (p = 0.612). Pairwise comparisons between multimodal monitoring, MEP only, and SSEP only were also not significant. Overall, specificity was consistently high, while most of the variation in diagnostic performance came from differences in sensitivity and false-negative rates.

**Table 2.** Pooled Diagnostic Accuracy of IONM

Outcome	Studies	Estimate	95% CI	95% prediction interval	I2 (%)	tau2
Sensitivity	36	90.1%	83.8% to 94.2%	43.4% to 99.1%	56.7	1.52
Specificity	38	95.4%	93.0% to 97.0%	62.1% to 99.6%	95.3	1.63
False-negative rate	36	9.9%	5.8% to 16.2%	0.9% to 56.6%	56.7	1.52
False-positive rate	38	4.6%	3.0% to 7.0%	0.4% to 37.9%	95.3	1.63
Diagnostic odds ratio	36	206.8	92.8 to 460.9	3.7 to 11615.1	73.9	4.06

***Note.*** Pooled estimates were generated using random-effects models with logit transformation. A continuity correction of 0.5 was applied to zero-cell counts. I^2^ values represent between-study heterogeneity, with higher values indicating greater inconsistency across included studies.

**Abbreviations:** CI, confidence interval; DOR, diagnostic odds ratio; FNR, false-negative rate; FPR, false-positive rate; IONM, intraoperative neurophysiologic monitoring; I^2^, Higgins inconsistency statistic.

**Figure 4.**
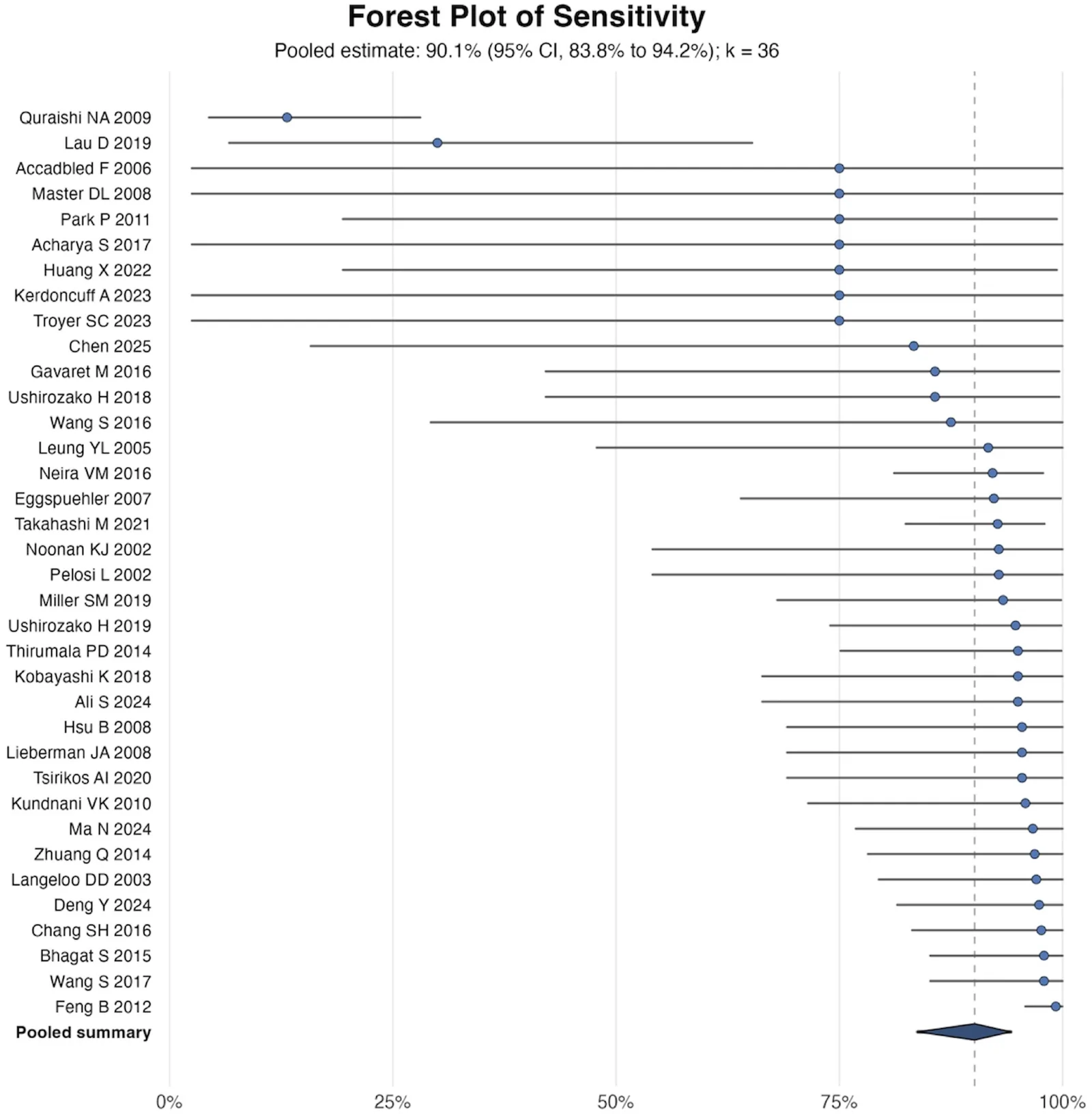
Forest plot of sensitivity across included studies using a random-effects logit model with exact 95% confidence intervals. Each point represents an individual study estimate, with horizontal lines indicating confidence intervals. The pooled estimate is shown as a summary point, demonstrating overall sensitivity of intraoperative neurophysiologic monitoring for detecting postoperative neurologic deficits

**Figure 5.**
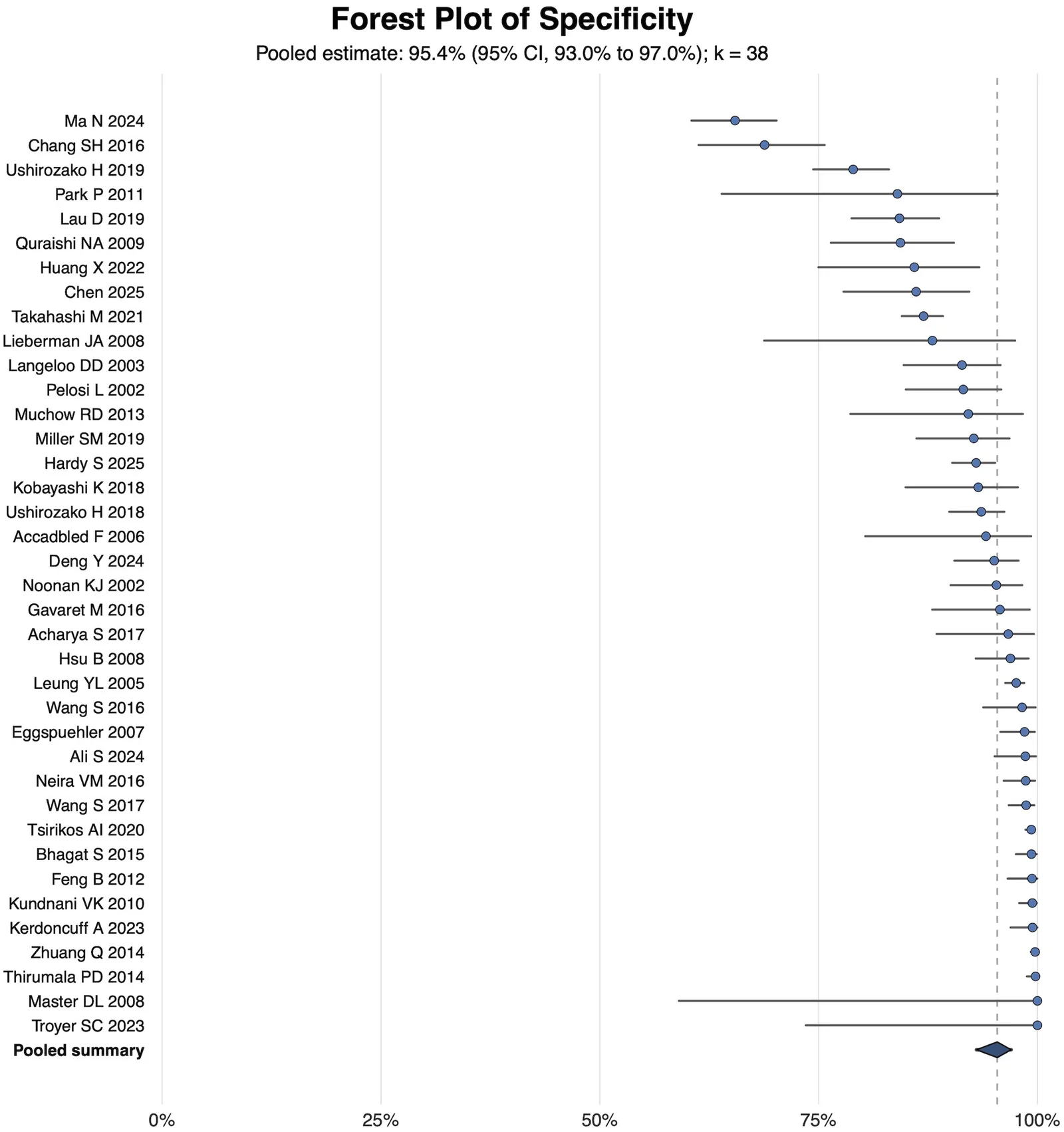
Forest plot of specificity across included studies using a random-effects logit model. Individual study estimates and corresponding confidence intervals are shown, with the pooled estimate indicating consistently high specificity of intraoperative neurophysiologic monitoring across studies

**Figure 6.**
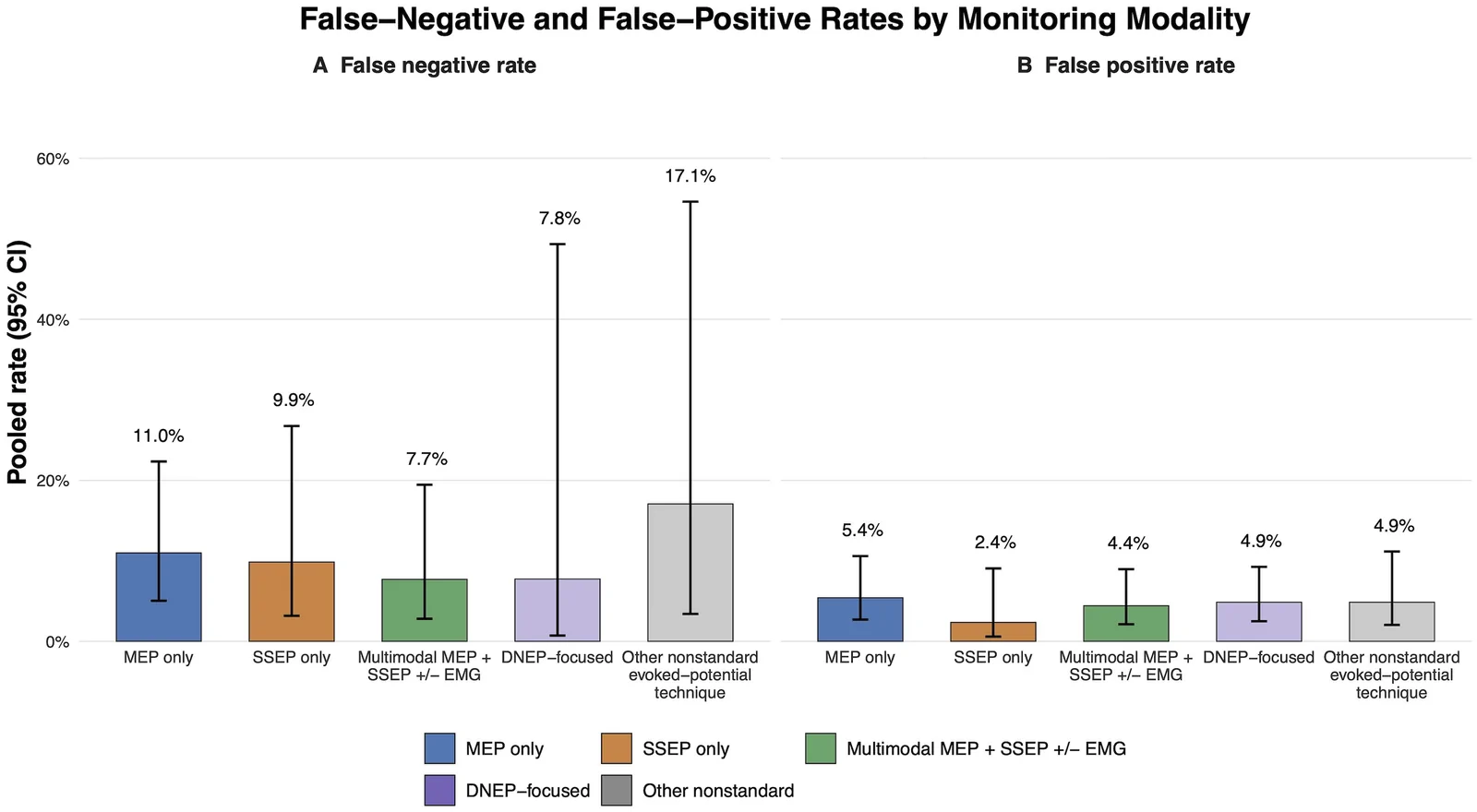
False-negative and false-positive rates by monitoring modality. Bar plots show pooled false-negative rates, defined as missed deficits, and pooled false-positive rates, defined as alerts without postoperative deficit, with 95% confidence intervals

### Summary Receiver Operating Characteristic Analysis

The SROC curve demonstrated strong overall diagnostic performance, with the pooled estimate located near the upper-left corner (Figure 7). The pooled sensitivity was 90.1% and the false-positive rate was 4.6% with 95% CIs of 83.8%-94.2% and 3.0%-7.0%, respectively. An exploratory funnel plot of the log diagnostic odds ratios was also generated to look for possible small-study effects (Supplemental Figure 2); no formal test for asymmetry was performed.

**Figure 7.**
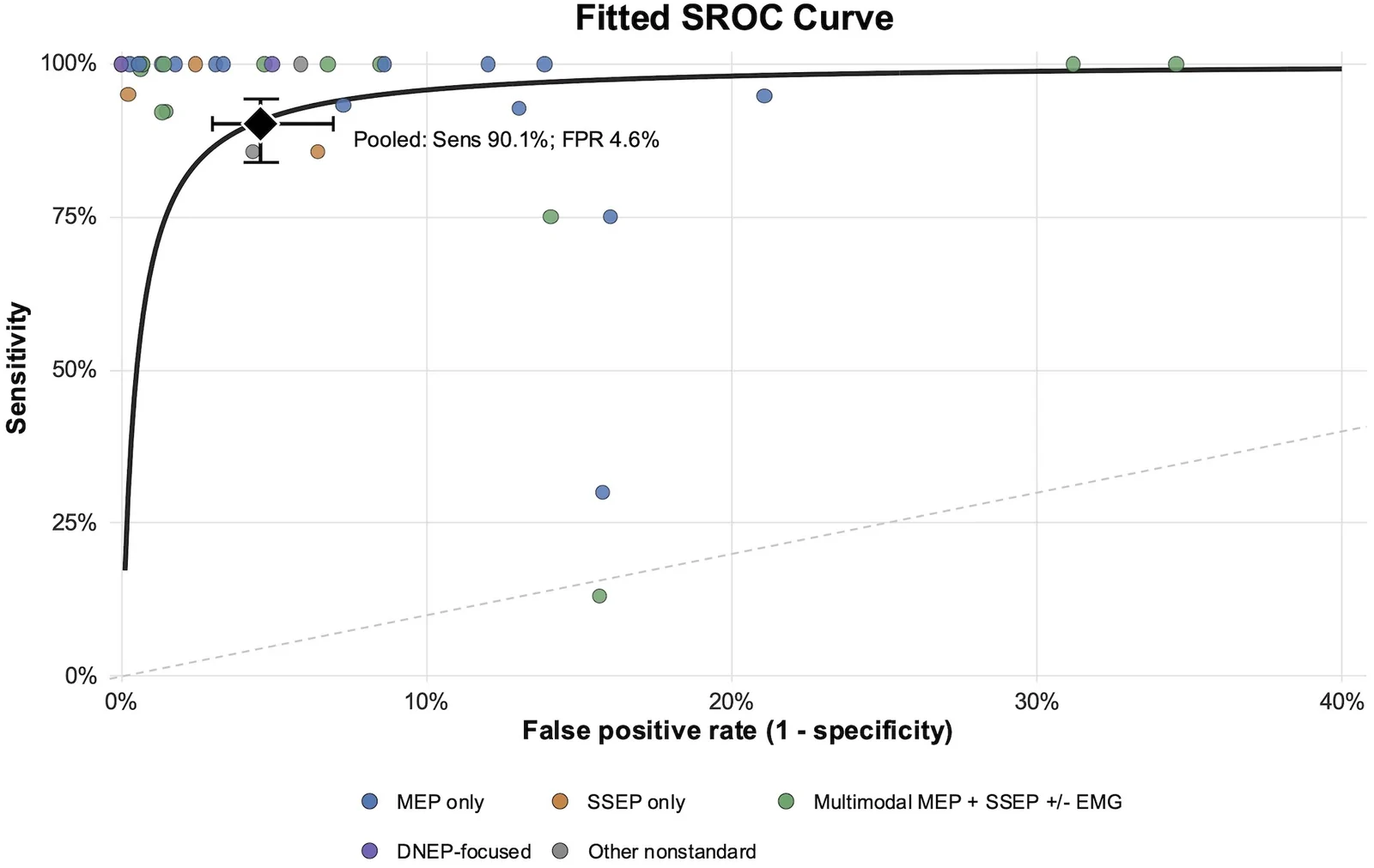
Summary receiver operating characteristic (SROC) curve demonstrating the overall diagnostic performance of intraoperative neurophysiologic monitoring. Each point represents an individual study, and the pooled estimate is located near the upper-left corner, reflecting high sensitivity and specificity across studies. The pooled point is shown at sensitivity 90.1% and false-positive rate 4.6%, with 95% CI bars for sensitivity (83.8% to 94.2%) and false-positive rate (3.0% to 7.0%)

False-negative and false-positive rates were also compared across monitoring modalities (Figure 8). Multimodal MEP + SSEP ± EMG had the lowest false-negative rate at 7.7%, followed by SSEP only at 9.9% and MEP only at 11.0%. The corresponding false-positive rates were 4.4%, 2.4%, and 5.4%. DNEP-focused and other nonstandard evoked-potential techniques were shown separately and were not included in the conventional modality meta-regression. None of the differences between the conventional monitoring groups were statistically significant.

**Figure 8.**
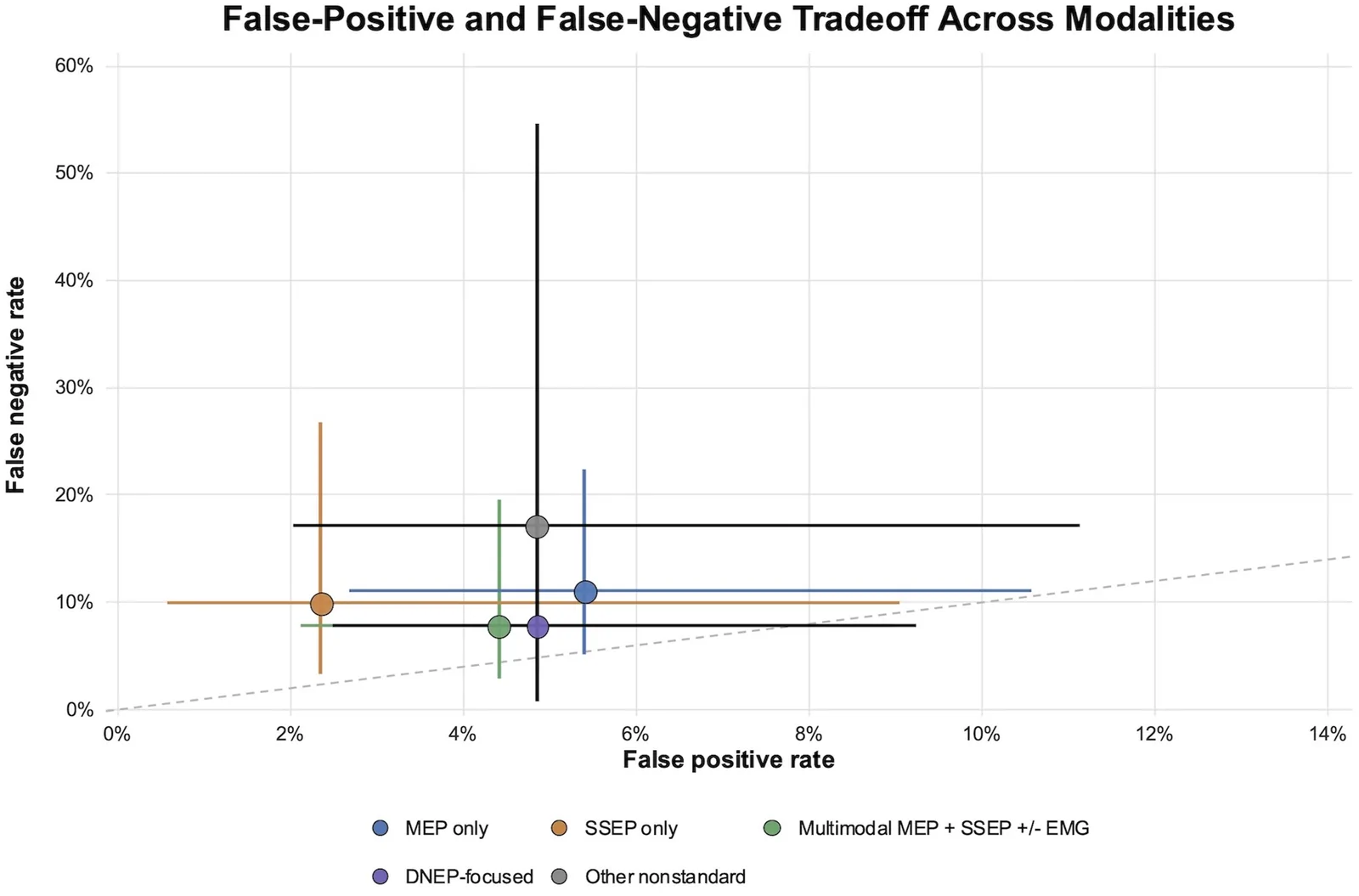
False-positive and false-negative rate tradeoff across monitoring modalities. Each point represents a modality-level pooled estimate, with horizontal and vertical bars indicating 95% confidence intervals for false-positive and false-negative rates, respectively. DNEP-focused and other nonstandard evoked-potential groups are displayed descriptively and were excluded from conventional modality meta-regression. Differences in sensitivity are primarily driven by variation in false-negative rates, while false-positive rates remain consistently low across modalities

### Subgroup Analysis by Monitoring Modality

Subgroup analysis demonstrated overlapping diagnostic performance across conventional monitoring modalities (Table 3). MEP only monitoring had a pooled sensitivity of 89.0% (95% CI, 77.7%-95.0%), and specificity of 94.6% (95% CI, 89.4%-97.3%). SSEP only monitoring had a sensitivity of 90.1% (95% CI, 73.2%-96.8%), and specificity was 97.6% (95% CI, 90.9%-99.4%). Multimodal MEP + SSEP ± EMG had a sensitivity of 92.3% (95% CI, 80.5%-97.2%) and specificity of 95.6% (95% CI, 91.0%-97.9%). None of these differences between modalities were statistically significant for either sensitivity (p = 0.902) or specificity (p = 0.612).

**Table 3.** Subgroup Analysis of Diagnostic Accuracy by Monitoring Modality

Monitoring Modality	Sensitivity	Specificity
MEP only	89.0% (77.7% to 95.0%)	94.6% (89.4% to 97.3%)
SSEP only	90.1% (73.2% to 96.8%)	97.6% (90.9% to 99.4%)
Multimodal MEP + SSEP +/- EMG	92.3% (80.5% to 97.2%)	95.6% (91.0% to 97.9%)

***Note.*** Sensitivity and specificity estimates are presented as pooled percentages with 95% confidence intervals. Between-group comparisons by monitoring modality were not statistically significant.

**Between-group p values:** sensitivity p = 0.902; specificity p = 0.612.

**Abbreviations:** CI, confidence interval; MEP, motor evoked potentials; SSEP, somatosensory evoked potentials; EMG, electromyography.

Figure 9 shows the study-level spread in sensitivity and specificity across monitoring modalities. Specificity remained high across the conventional groups, while sensitivity varied more from study to study. DNEP-focused and other nonstandard evoked-potential techniques are shown separately for context and were not included in the conventional modality meta-regression.

**Figure 9.**
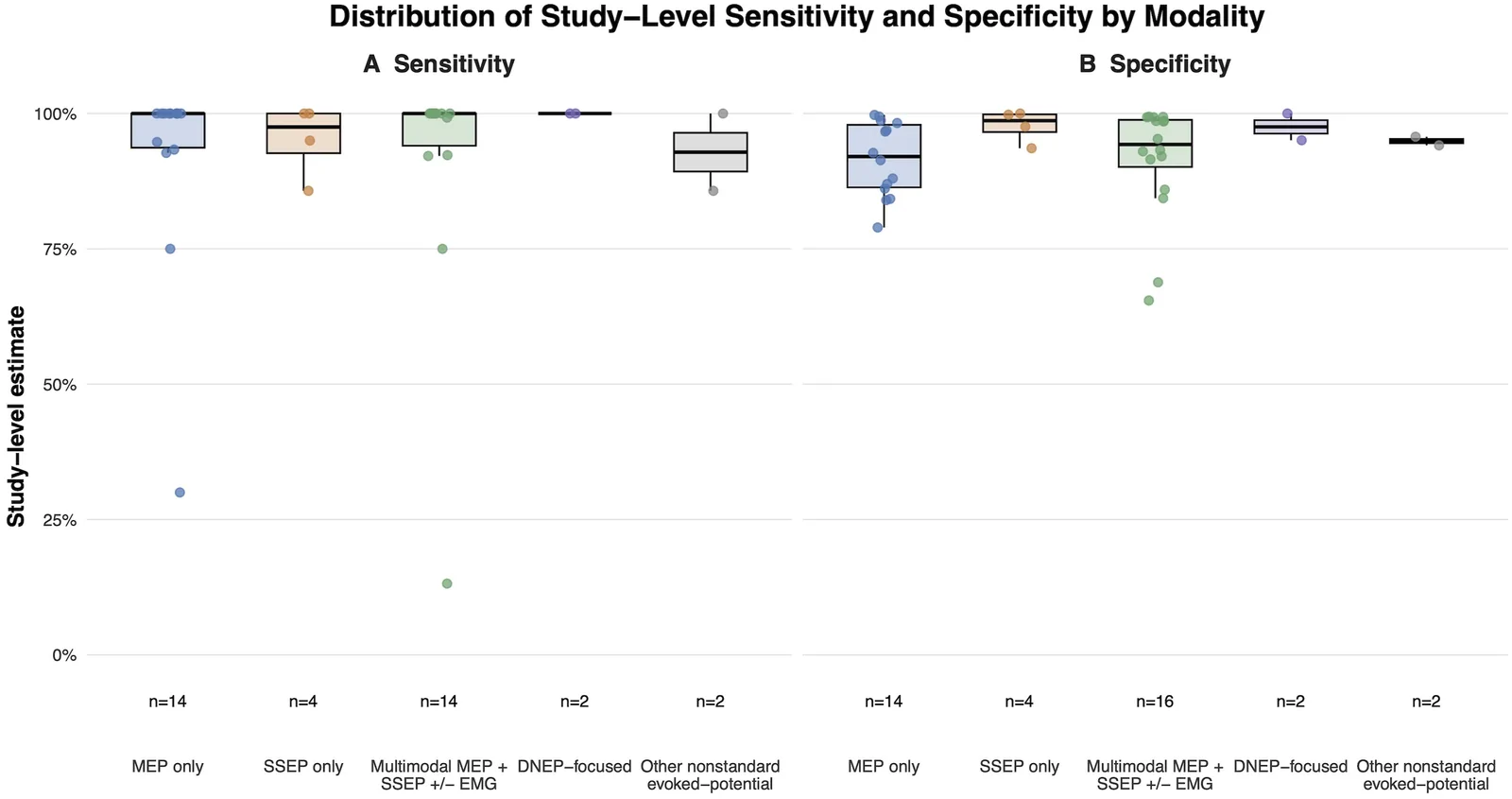
Distribution of study-level sensitivity and specificity estimates stratified by monitoring modality. Box-and-whisker plots display median values and interquartile ranges, illustrating modality-specific variability in study-level diagnostic performance

### Study Size and Diagnostic Performance

Evaluation of study size in relation to diagnostic performance (Figure 10) demonstrated no consistent relationship between sample size and sensitivity or specificity. High sensitivity estimates were observed across both small and large cohorts, and variability persisted regardless of study size. These findings suggest that heterogeneity in IONM performance is not primarily driven by sample size but instead reflects differences in monitoring protocols, patient populations, and intraoperative factors.

**Figure 10.**
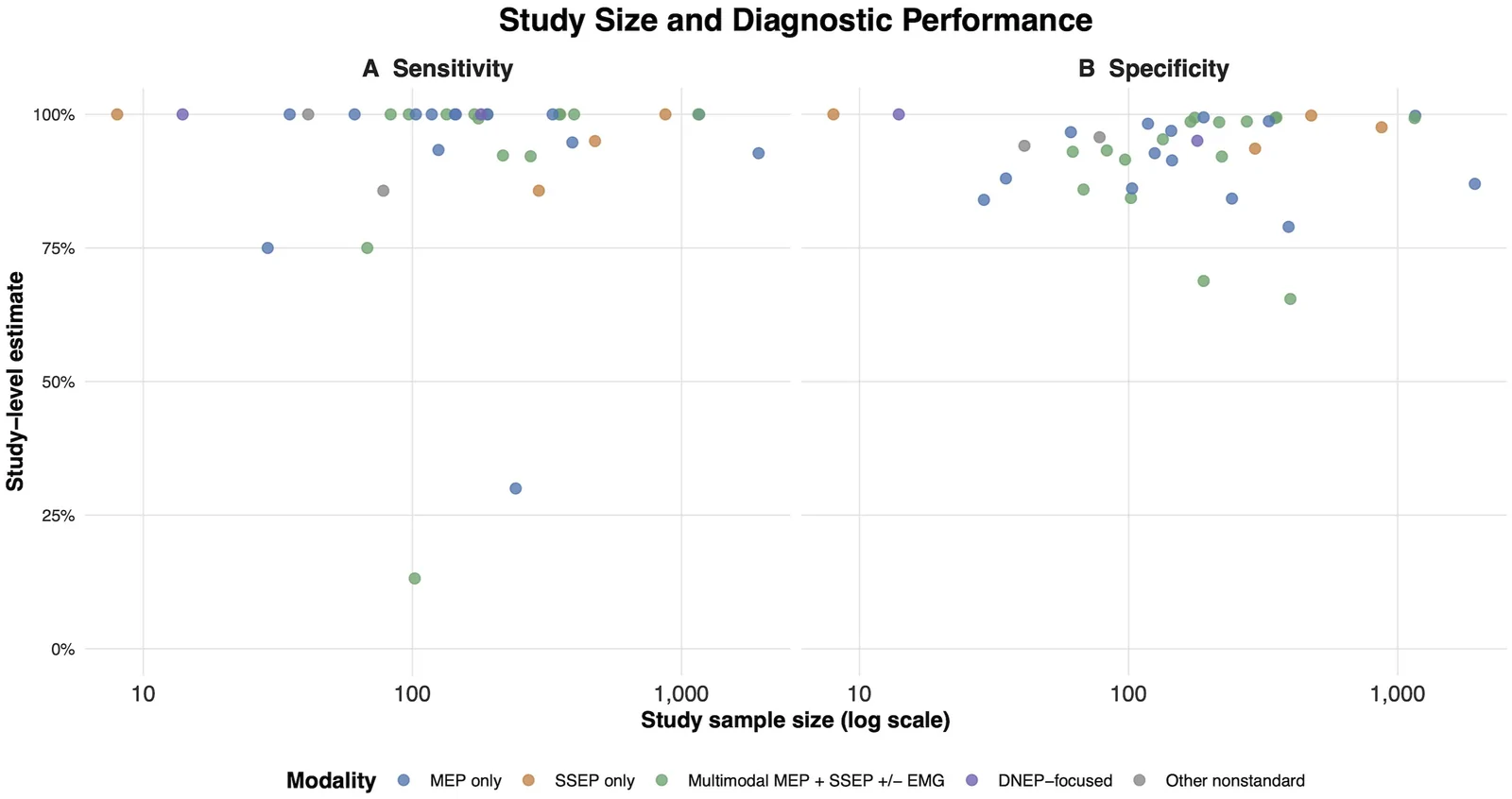
Relationship between study sample size and diagnostic performance. Each point represents an individual study plotted against sensitivity and specificity. No clear association is observed, indicating that variability in diagnostic accuracy is not primarily driven by study size but likely reflects differences in monitoring protocols and intraoperative factors

## Summary of Findings

Across the included studies, IONM had a pooled sensitivity of 90.1% and specificity of 95.4%. The pooled false-negative rate was 9.9%, while the false-positive rate was 4.6%. The false-negative rate remains clinically important because it represents postoperative deficits that occurred without a preceding monitoring alert. Multimodal monitoring had the highest pooled sensitivity among the conventional approaches, but the differences between modalities were not statistically significant. Although the pooled results were favorable, performance varied considerably across studies, showing the need for more consistent alert thresholds and postoperative reporting.

## Discussion

This systematic review and meta-analysis represents one of the largest investigations of IONM accuracy in spinal deformity surgery. This analysis showed a high pooled sensitivity (90.1%) and specificity (95.4%) in predicting post-operative deficits (Figures 4 and 5). Furthermore, the high diagnostic odds ratio of 206.8 confirms IONM’s potential for differentiating true neurologic compromise in real-time (Table 2). Overall, the results of this study support IONM as a valuable tool for detecting intraoperative neurologic perturbation during complex spinal deformity surgery, while highlighting important differences in how monitoring modalities should be interpreted.^2,4^

These results are consistent with those of other meta-analyses conducted to evaluate the efficacy of IONM in spine surgery, which showed high specificity, but varied sensitivity based on modality. For example, Thirumala et al showed pooled sensitivity for SSEPs to be approximately 71%, with specificity consistently over 95%, whereas for MEPs, sensitivities as high as 90% in detecting postoperative deficits.^9^ In the present analysis, sensitivity estimates were broadly comparable across multimodal, MEP-only, and SSEP-only monitoring, with multimodal monitoring demonstrating the highest pooled sensitivity descriptively at 92.3%. However, confidence intervals overlapped, and between-modality differences were not statistically significant. The pooled specificity across all modalities remained consistently high, exceeding 91%. These descriptive differences between modalities may reflect the neurophysiologic pathways assessed by each modality, as MEPs directly monitor corticospinal tract function while SSEPs monitor dorsal column integrity, which may be less sensitive for isolated motor deficits.^8,9,11^ However, the absence of statistically significant differences between modalities emphasizes the importance of interpreting these findings cautiously. Most studies did not describe false-negative deficits in enough detail to determine whether they were predominantly motor, sensory, cord-level, or root-level.

Clinically, our results support the common practice of utilizing multimodal monitoring in complex cord level spine deformity surgery, as both ascending and descending pathways are at risk. Importantly, this study builds on prior literature in that we specifically looked at spine deformity surgery, allowing for a more detailed examination of modality performance based on a larger pooled patient group. However, the consistency of IONM’s performance across studies remains variable, suggesting that these findings must be interpreted within a broader clinical context particularly given that diagnostic accuracy metrics do not fully capture the dynamic and intervention-dependent nature of intraoperative monitoring.

The heterogeneity in study findings was significant with regard to sensitivity; however, it is not a new observation in the overall body of work in deformity surgery.^58^ The degree of deformity, type of surgery, surgery technique, anesthetic management, level of surgery and alert thresholds likely contribute to the variation reported.^59,60^ The lack of consistency in the definition of neurologic outcomes can be considered an added source of heterogeneity.^61^ This variability may obscure clinically meaningful differences in IONM performance at the patient and procedure level.

Most studies used TIVA (24/43), while only 2 used inhalational anesthesia and 9 used mixed or transitioned regimens; 8 did not report enough detail to classify the regimen. Because anesthesia reporting was incomplete and closely related to monitoring modality and study design, these findings were summarized descriptively rather than compared formally. Alert criteria also varied across studies, reinforcing the need for clearly defined, prospectively validated, modality-specific thresholds.

When IONM is utilized in surgery it should be interpreted as an intervention dependent monitoring system rather than a traditional diagnostic test.^62^ Intraoperative signals often prompt the surgeon to intervene, whether it is reversing the previous maneuver, adjustment of instrumentation, optimization of hemodynamics, or adjustment of anesthetic depth to prevent potential neurologic compromise leading to permanent injury.^63^ This dynamic relationship intraoperatively can complicate the decision making of the surgeon when an alert is sounded. The team considers if the signal is a true positive that could compromise fragile neurologic tissue as all alerts may not result in postoperative deficits due to successful intraoperative intervention.^64,65^ With this context in mind, special consideration of MEP interpretation must be given. Although MEP monitoring remains highly sensitive and clinically important, MEP signals are susceptible to anesthetic effects and physiologic variability, which may contribute to transient changes that do not reflect true neurologic injury.^18,66,67^ As a result, distinguishing clinically meaningful alerts from reversible intraoperative perturbations remains a key challenge. Therefore, while MEP monitoring improves detection of neurologic perturbation, it may also increase sensitivity to reversible physiologic changes, requiring careful clinical interpretation to distinguish true neurologic injury from transient intraoperative effects.

These findings reveal an important gap in the current literature. While IONM yields strong overall diagnostic performance, there remains limited understanding of how intraoperative signal changes, particularly MEP alerts, translate to patient-level neurologic outcomes, especially in the context of intraoperative interventions that may alter the natural course of neurologic injury. Future studies using standardized definitions and patient-level data will be essential to better characterize signal reliability and refine intraoperative decision making in spinal deformity surgery.

## Limitations

This study has several limitations. First, the majority of included studies were retrospective in nature, which introduces potential bias related to patient selection, outcome reporting, and variation in follow-up. Second, for the diagnostic accuracy analysis, only studies that had sufficient data were included to construct diagnostic contingency tables. This potentially introduces selection bias and could limit generalizability. Some studies reported diagnostic outcomes by procedure rather than by patient. Patients undergoing repeated procedures may therefore have contributed more than one observation, and the published aggregate data did not allow us to account for within-patient correlation. Third, considerable heterogeneity exists between studies as a result of the varying severity of the deformities, surgical approaches, anesthetic techniques, and IONM alert criteria that could not be fully standardized. Furthermore, variation in neurologic outcome definitions and preoperative and postoperative deficit thresholds may affect diagnostic performance. Additionally, monitoring cord level injuries seen in complex spinal deformity may have higher sensitivity and specificity compared to root level degenerative surgery which may bias IONM performance in this study. Lastly, although PubMed, Embase, and Web of Science were searched through May 1, 2026, additional databases, unpublished sources, or studies published after the search date may still contain relevant data.

## Conclusion

In conclusion, this systematic review and meta-analysis shows the utility IONM possesses intraoperatively. It demonstrates a strong diagnostic performance in spinal deformity surgery, characterized by high specificity and favorable sensitivity across monitoring modalities. These results support the continued practice of employing this important tool during cord level complex spinal surgeries to help mitigate neurologic risk and permanent deficits postoperatively. However, variability in sensitivity and intraoperative factors, particularly affecting MEP signals, complicate interpretation of alerts, emphasizing the need for improved standardization and patient-level analyses to better define clinically meaningful signal changes. Future studies should use clearly defined, prospectively validated alert thresholds and consistently report anesthetic regimens and patient-level outcomes to improve interpretation of IONM findings across spinal deformity surgery.

## Supplemental Material

Supplemental Material - Diagnostic Accuracy of Intraoperative Neurophysiologic Monitoring in Spinal Deformity Surgery: Systematic Review and Meta-Analysis Supplemental Material for Diagnostic Accuracy of Intraoperative Neurophysiologic Monitoring in Spinal Deformity Surgery: Systematic Review and Meta-Analysis by Brendan T. O’Reilly, Alexandra M. Dunker, Drue M. Domagala, Allie C. Reynolds, Catalina Garcia, Sohan Arakal, Giselle K. Henry, David Travis Johnston, Rajiv S. Dharnipragada, Stavros Matsoukas, Theodore Gallas, Noah C. Fulcomer, Shyon K. Ahmadi, Alexander Lee and Eric O. Klineberg in Global Spine Journal.

Supplemental Material - Diagnostic Accuracy of Intraoperative Neurophysiologic Monitoring in Spinal Deformity Surgery: Systematic Review and Meta-Analysis Supplemental Material for Diagnostic Accuracy of Intraoperative Neurophysiologic Monitoring in Spinal Deformity Surgery: Systematic Review and Meta-Analysis by Brendan T. O’Reilly, Alexandra M. Dunker, Drue M. Domagala, Allie C. Reynolds, Catalina Garcia, Sohan Arakal, Giselle K. Henry, David Travis Johnston, Rajiv S. Dharnipragada, Stavros Matsoukas, Theodore Gallas, Noah C. Fulcomer, Shyon K. Ahmadi, Alexander Lee and Eric O. Klineberg in Global Spine Journal.

Supplemental Material - Diagnostic Accuracy of Intraoperative Neurophysiologic Monitoring in Spinal Deformity Surgery: Systematic Review and Meta-Analysis Supplemental Material for Diagnostic Accuracy of Intraoperative Neurophysiologic Monitoring in Spinal Deformity Surgery: Systematic Review and Meta-Analysis by Brendan T. O’Reilly, Alexandra M. Dunker, Drue M. Domagala, Allie C. Reynolds, Catalina Garcia, Sohan Arakal, Giselle K. Henry, David Travis Johnston, Rajiv S. Dharnipragada, Stavros Matsoukas, Theodore Gallas, Noah C. Fulcomer, Shyon K. Ahmadi, Alexander Lee and Eric O. Klineberg in Global Spine Journal.

## Data Availability

The data extracted from included studies and analytic code are available from the corresponding author upon reasonable request.*
