# Supplementary material for: Diagnostic Accuracy of Intraoperative Neurophysiologic Monitoring in Spinal Deformity Surgery: Systematic Review and Meta-Analysis
Source: Global Spine J. 2026 Sep 28:21925682261492292. Online ahead of print. doi: 10.1177/21925682261492292 (PMC13619987; doi:10.1177/21925682261492292)
Supplement: Supplementary file 1 [file sj-pdf-1-gsj-10.1177_21925682261492292.pdf]

Supplementary Appendix 1. Full Electronic Search Strategy

Diagnostic Accuracy of Intraoperative Neurophysiologic Monitoring in Spinal Deformity Surgery: Systematic Review and Meta-Analysis

1. Overview

A systematic electronic search was performed to identify studies evaluating intraoperative neurophysiologic monitoring in spinal deformity surgery. The strategy was designed to capture literature addressing diagnostic accuracy outcomes relevant to intraoperative monitoring performance. Search terms combined controlled vocabulary and keyword terms, where applicable, related to monitoring modality, spinal deformity diagnoses, and diagnostic accuracy measures.

2. Databases, Date Limits, and Records Identified

Database	Records identified	Years searched	Search concepts
PubMed	128	2000–2026	IONM / MEP / SSEP; spinal deformity / scoliosis / kyphosis; sensitivity, specificity, accuracy, predictive value
Embase	185	2000–2026	IONM / MEP / SSEP; spinal deformity / scoliosis / kyphosis; sensitivity, specificity, accuracy, predictive value
Web of Science Core Collection	160	2000–2026	IONM / MEP / SSEP; spinal deformity / scoliosis / kyphosis; sensitivity, specificity, accuracy, predictive value
**Total**	**473**	**January 1, 2000–May 1, 2026**	**All database records before duplicate removal**

Date limits were applied within each database interface. Database-specific syntax was used while preserving the same core concepts across PubMed, Embase, and Web of Science.

3. PubMed Search Strategy

Database: PubMed. Years searched: 2000–2026. Records identified: 128.

One-line executable PubMed search string

```
("Monitoring, Intraoperative"[Mesh] OR "Evoked Potentials, Motor"[Mesh] OR "Evoked Potentials,
Somatosensory"[Mesh] OR "intraoperative neurophysiologic monitoring"[tiab] OR IONM[tiab] OR "motor
evoked potential*"[tiab] OR MEP[tiab] OR "somatosensory evoked potential*"[tiab] OR SSEP[tiab] OR
neuromonitor*[tiab]) AND ("Spinal Curvatures"[Mesh] OR "Scoliosis"[Mesh] OR "Kyphosis"[Mesh] OR spinal
deformity[tiab] OR scoliosis[tiab] OR kyphosis[tiab] OR "Scheuermann* kyphosis"[tiab] OR "adolescent
idiopathic scoliosis"[tiab] OR AIS[tiab]) AND (sensitivity[tiab] OR specificity[tiab] OR
accuracy[tiab] OR "predictive value"[tiab])
```

Readable line-broken PubMed search string

```
(
"Monitoring, Intraoperative"[Mesh]
OR "Evoked Potentials, Motor"[Mesh]
OR "Evoked Potentials, Somatosensory"[Mesh]
OR "intraoperative neurophysiologic monitoring"[tiab]
OR IONM[tiab]
OR "motor evoked potential*"[tiab]
OR MEP[tiab]
OR "somatosensory evoked potential*"[tiab]
OR SSEP[tiab]
OR neuromonitor*[tiab]
)
AND
(
"Spinal Curvatures"[Mesh]
OR "Scoliosis"[Mesh]
OR "Kyphosis"[Mesh]
OR spinal deformity[tiab]
OR scoliosis[tiab]
OR kyphosis[tiab]
OR "Scheuermann* kyphosis"[tiab]
OR "adolescent idiopathic scoliosis"[tiab]
OR AIS[tiab]
)
AND
(
sensitivity[tiab]
OR specificity[tiab]
OR accuracy[tiab]
OR "predictive value"[tiab]
)
```

4. Embase Search Strategy

Database: Embase. Years searched: 2000–2026. Records identified: 185.

One-line executable Embase search string

```
('intraoperative neurophysiologic monitoring':ti,ab OR 'intraoperative neuromonitoring':ti,ab OR ionm:ti,ab OR neuromonitor*:ti,ab OR 'motor evoked potential*':ti,ab OR mep:ti,ab OR 'somatosensory evoked potential*':ti,ab OR ssep:ti,ab) AND ('spinal deformity':ti,ab OR scoliosis:ti,ab OR kyphosis:ti,ab OR 'adolescent idiopathic scoliosis':ti,ab OR ais:ti,ab OR 'adult spinal deformity':ti,ab OR 'Scheuermann* kyphosis':ti,ab) AND (sensitivity:ti,ab OR specificity:ti,ab OR accuracy:ti,ab OR 'predictive value':ti,ab)
```

Readable line-broken Embase search string

```
(
'intraoperative neurophysiologic monitoring':ti,ab
OR 'intraoperative neuromonitoring':ti,ab
OR ionm:ti,ab
OR neuromonitor*:ti,ab
OR 'motor evoked potential*':ti,ab
OR mep:ti,ab
OR 'somatosensory evoked potential*':ti,ab
OR ssep:ti,ab
)
AND
(
'spinal deformity':ti,ab
OR scoliosis:ti,ab
OR kyphosis:ti,ab
OR 'adolescent idiopathic scoliosis':ti,ab
OR ais:ti,ab
OR 'adult spinal deformity':ti,ab
OR 'Scheuermann* kyphosis':ti,ab
)
AND
(
sensitivity:ti,ab
OR specificity:ti,ab
OR accuracy:ti,ab
OR 'predictive value':ti,ab
)
```

5. Web of Science Search Strategy

Database: Web of Science Core Collection. Years searched: 2000–2026. Records identified: 160.

One-line executable Web of Science search string

```
(IONM OR MEP OR SSEP OR "intraoperative neurophysiologic monitoring" OR neuromonitor*) AND (scoliosis
OR kyphosis OR "spinal deformity" OR AIS OR "adult spinal deformity") AND (sensitivity OR specificity
OR accuracy OR "predictive value")
```

Readable line-broken Web of Science search string

```
(
IONM
OR MEP
OR SSEP
OR "intraoperative neurophysiologic monitoring"
OR neuromonitor*
)
AND
(
scoliosis
OR kyphosis
OR "spinal deformity"
OR AIS
OR "adult spinal deformity"
)
AND
(
sensitivity
OR specificity
OR accuracy
OR "predictive value"
)
```

6. Notes

- The PubMed search incorporated MeSH terms and title/abstract keyword terms. Embase and Web of Science strategies were adapted from the PubMed framework using database-specific syntax.
- The search was limited to studies published from January 1, 2000, through May 1, 2026. Year filters were applied within the database interfaces where appropriate.
- The search concepts were monitoring modality, spinal deformity pathology, and diagnostic accuracy outcomes. Records identified across databases were subsequently deduplicated and screened according to the eligibility criteria described in the manuscript.
