# Supplementary material for: Diagnostic Accuracy of Intraoperative Neurophysiologic Monitoring in Spinal Deformity Surgery: Systematic Review and Meta-Analysis
Source: Global Spine J. 2026 Sep 28:21925682261492292. Online ahead of print. doi: 10.1177/21925682261492292 (PMC13619987; doi:10.1177/21925682261492292)
Supplement: Supplementary file 2 [file sj-pdf-2-gsj-10.1177_21925682261492292.pdf]

Supplementary Table 1. Study-Level Diagnostic Accuracy Data

Study	TP	FP	TN	FN	Sensitivity	Specificity
Accadbled F 2006[22]	1	2	32	0	1.00	0.94
Acharya S 2017[40]	1	2	58	0	1.00	0.97
Ali S 2024[52]	9	2	143	0	1.00	0.99
Bhagat S 2015[34]	23	2	290	0	1.00	0.99
Chang SH 2016[36]	20	53	117	0	1.00	0.69
Chen 2025[55]	2	14	87	0	1.00	0.86
Deng Y 2024[51]	18	8	154	0	1.00	0.95
Eggspuehler 2007[23]	12	3	201	1	0.92	0.99
Feng B 2012[30]	130	1	160	1	0.99	0.99
Gavaret M 2016[37]	6	3	67	1	0.86	0.96
Hardy S 2025[54]	0	32	425	0	Not estimable	0.93
Hsu B 2008[26]	10	5	157	0	1.00	0.97
Huang X 2022[47]	3	9	55	1	0.75	0.86
Kerdoncuff A 2023[49]	1	1	178	0	1.00	0.99
Kobayashi K 2018[41]	9	5	69	0	1.00	0.93
Kundnani VK 2010[28]	11	2	341	0	1.00	0.99
Langeloo DD 2003[20]	16	10	106	0	1.00	0.91
Lau D 2019[43]	3	35	187	7	0.30	0.84
Leung YL 2005[21]	5	21	845	0	1.00	0.98
Lieberman JA 2008[24]	10	3	22	0	1.00	0.88
Ma N 2024[53]	14	133	252	0	1.00	0.65
Master DL 2008[25]	1	0	7	0	1.00	1.00
Miller SM 2019[42]	14	8	102	1	0.93	0.93
Muchow RD 2013[31]	0	3	35	0	Not estimable	0.92
Neira VM 2016[38]	47	3	221	4	0.92	0.99
Noonan KJ 2002[19]	6	6	122	0	1.00	0.95
Park P 2011[29]	3	4	21	1	0.75	0.84
Pelosi L 2002[18]	6	10	108	0	1.00	0.92
Quraishi NA 2009[27]	5	18	97	33	0.13	0.84
Takahashi M 2021[45]	51	108	722	4	0.93	0.87
Thirumala PD 2014[32]	19	1	457	1	0.95	1.00
Troyer SC 2023[50]	1	0	12	0	1.00	1.00
Tsirikos AI 2020[7]	10	8	1,137	0	1.00	0.99
Ushirozako H 2018[13]	6	17	248	1	0.86	0.94
Ushirozako H 2019[11]	18	76	285	1	0.95	0.79
Wang S 2016[35]	3	2	112	0	1.00	0.98
Wang S 2017[39]	23	4	305	0	1.00	0.99
Zhuang Q 2014[33]	15	3	1,144	0	1.00	1.00

Note. Study-level diagnostic accuracy data are shown for the 38 analyzable studies contributing to the pooled diagnostic accuracy meta-analysis. Raw sensitivity and specificity were calculated directly from reconstructed TP, FP, TN, and FN values. Studies with TP + FN = 0 had no postoperative neurologic-deficit-positive cases; sensitivity and false-negative rate were therefore not estimable. Continuity corrections were used only where required for model fitting or forest-plot display, not to redefine raw study-level diagnostic accuracy.

Abbreviations: TP, true positive; FP, false positive; TN, true negative; FN, false negative.
