# Supplementary material for: Diagnostic Accuracy of Intraoperative Neurophysiologic Monitoring in Spinal Deformity Surgery: Systematic Review and Meta-Analysis
Source: Global Spine J. 2026 Sep 28:21925682261492292. Online ahead of print. doi: 10.1177/21925682261492292 (PMC13619987; doi:10.1177/21925682261492292)

Supplementary Figure 1: Study-level diagnostic accuracy data across the 38 studies with constructable 2 × 2 tables. Sensitivity is shown for the 36 studies in which it could be calculated, while specificity is shown for all 38 studies.

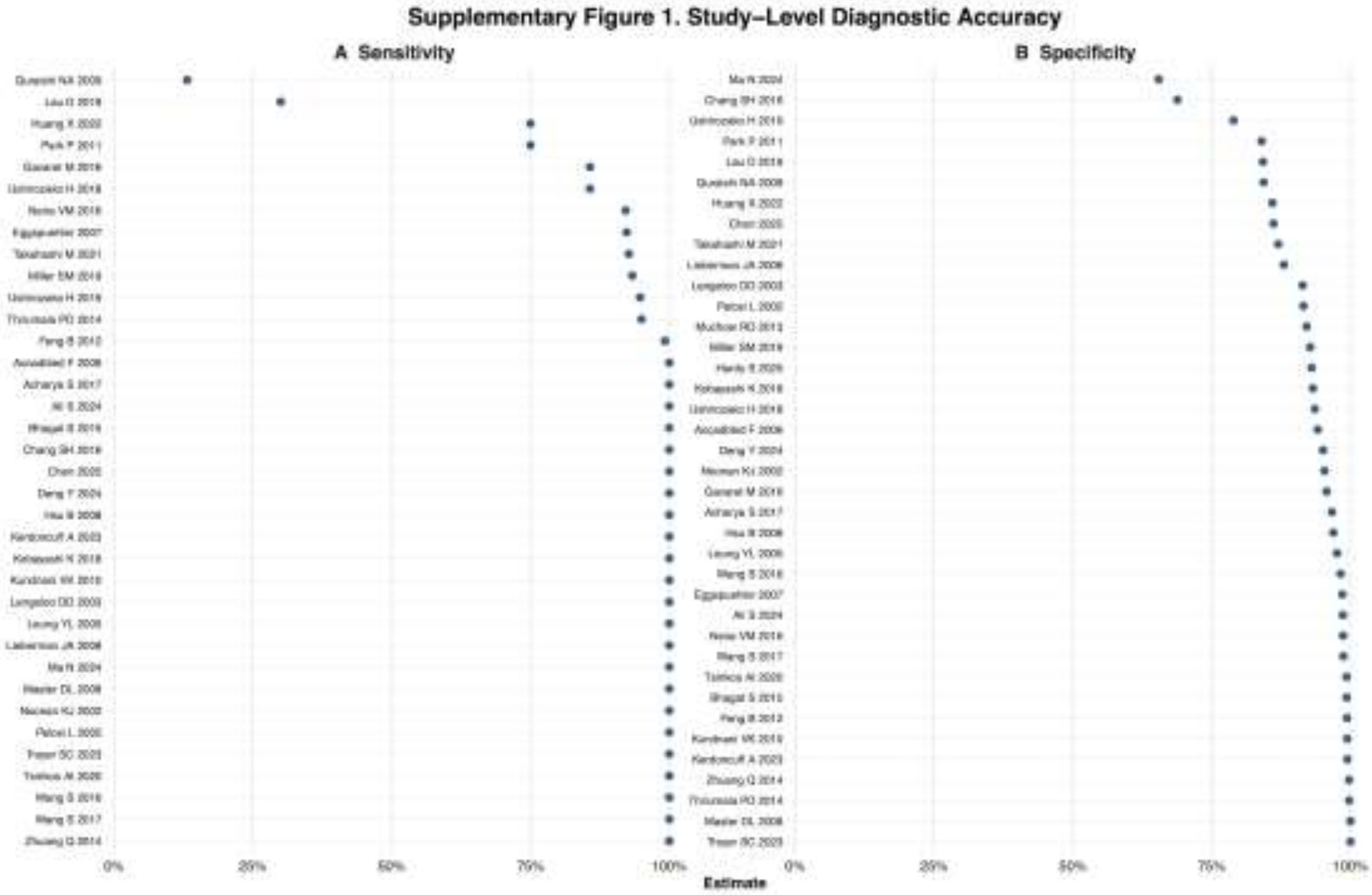

Supplementary Figure 2: Exploratory funnel plot of log diagnostic odds ratios. Each point represents an individual study included in the pooled diagnostic accuracy meta-analysis. The vertical dashed line indicates the pooled log diagnostic odds ratio, and the diagonal lines represent pseudo-95% confidence limits.

Supplementary Figure 2. Exploratory Funnel Plot of Log Diagnostic Odds Ratios

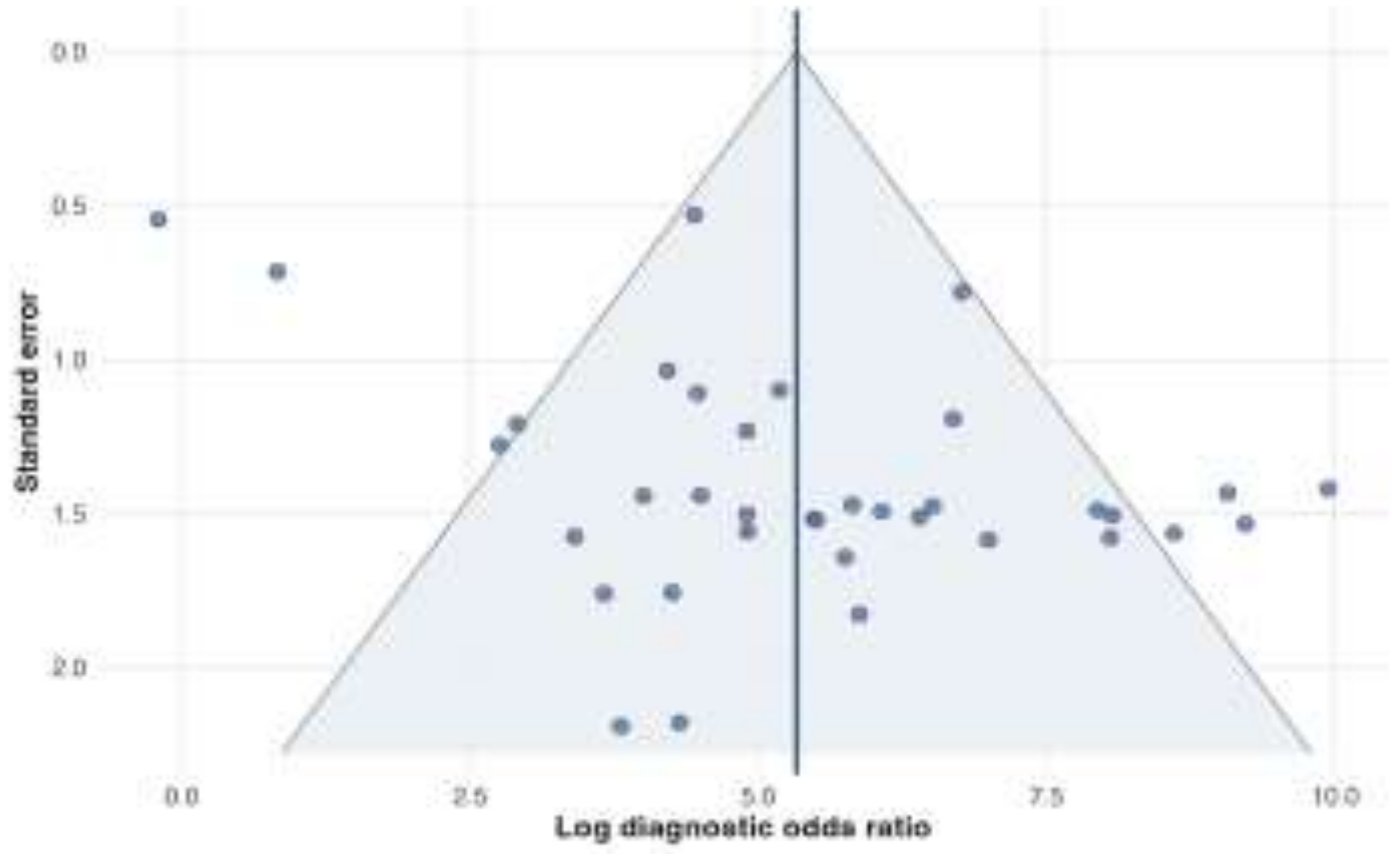

Supplement: Supplementary file 3 [file sj-pdf-3-gsj-10.1177_21925682261492292.pdf]
